# New circulating biomarkers for predicting cardiovascular death in healthy population

**DOI:** 10.1111/jcmm.12652

**Published:** 2015-08-10

**Authors:** Olle Melander, Javier Modrego, Jose J Zamorano-León, Juana M Santos-Sancho, Vicente Lahera, Antonio J López-Farré

**Affiliations:** a Department of Clinical Sciences, Lund UniversityMalmö, Sweden; b Instituto de Investigacion Sanitaria del Hospital Clínico San Carlos (IdISSC)Madrid, Spain; c Department of Preventive Medicine and Public Health, School of Medicine, Universidad ComplutenseMadrid, Spain; d Department of Physiology, School of Medicine, Universidad ComplutenseMadrid, Spain; e Department of Medicine, School of Medicine, Universidad ComplutenseMadrid, Spain

**Keywords:** cardiovascular death, plasma biomarkers, proteomics, risk factors

## Abstract

There is interest to analyse newer biomarkers to identify healthy individuals at risk to develop cardiovascular disease (CVD) incidents and death. To determine in healthy individuals new circulating protein biomarkers, whose systemic levels may be associated with the risk of future development of CVD incidents and death. The study was performed in 82 individuals from the Malmö Diet and Cancer study cohort, free from CVD of whom 41 developed CVD and 41 did not. Plasma proteins related to inflammation and thrombo-coagulating processes were analysed. α1-antitrypsin isotype 3 plasma levels were significantly higher while apolipoprotein J plasma levels were lower in participants that developed CVD incidents than those that did not develop acute cardiovascular episode. Of 82 participants, 17 died by CVD causes. There were proteins whose expression in plasma was significantly higher in participants suffering CVD death as compared with those that did not die by CVD. These proteins included: fibrinogen β-chain isotypes 1 and 3, fibrinogen-γ-chain isotype 2, vitamin D-binding protein isotypes 1, 2 and 3, α1-antitrypsin isotypes 3 and 6, haptoglobin isotypes 3,4,5 and 5, haemopexin isotypes 1 and 2, and Rho/Rac guanine nucleotide exchange factor 2. Moreover, apolipoprotein J plasma levels were found lower in participants that died by cardiovascular cause. Association between plasma levels of proteins and CVD death was independent of age, gender, conventional risk factors and plasma C-reactive protein levels. Several protein plasma levels and protein isotypes related to inflammation and thrombo-coagulating phenomena were independently associated with the risk of future CVD death.

## Introduction

Cardiovascular disease (CVD) is the major cause of premature death in Europe and is an important cause to decrease the quality of life and disability and contributes to the increase in the costs of health care 1. Therefore, to increase both the benefits of health and cost effectiveness, it is important to enhance the accuracy of the identification of the individuals at risk for CVD.

Cardiovascular morbidity and mortality in European and industrialized countries, is mainly associated with acute cerebrovascular and coronary arterial events 2. A large proportion of individuals with cardiovascular events have one or fewer of the conventional risk factors that include smoking, diabetes, hypertension or hyperlipidaemia 3. However, other individuals will never develop acute cardiovascular incidents although they have the same conventional risk factors. This suggests that, at least for some type of patients, these conventional risk factors, might not fully explain the risk of developing an acute cardiovascular event. As a result, in recent years, an increased interest has been growing to find and identify new molecular mechanisms associated with the genesis of acute cardiovascular events. The identification of these new biomarkers related to these novel molecular mechanisms may improve the prediction of cardiovascular events and death.

Inflammation as well as platelet hyperaggregability are main mechanisms leading to cardiovascular incidents 4,5. Indeed, systemic biomarkers of both inflammation and thrombo-coagulation have been proposed as potentially useful for the early detection of the risk of cardiovascular incidents in both apparently healthy individuals and patients with CVD 6,7. However, controversial results have been obtained using such biomarkers. An example is C-reactive protein, where some reports indicated predictable risks while other authors have obtained little information through C-reactive protein determination 8,9.

Several factors may influence the differing conclusions regarding the utility of such biomarkers as predictors of cardiovascular risk. Among them, the study population and the high specificity of the selected biomarkers may influence how well the biomarkers predict outcome. In this regard, another important consideration is the selection of the biomarkers. It is probable that the non-conclusive results may be influenced by the fact that most of the studies were based on the simultaneous determination of only a reduced number of informative biomarkers. In addition, it is also likely that other circulating proteins and protein isotypes, not previously studied in relation to prediction of cardiovascular incidents and cardiovascular death, may exist. For this reason, there is substantial interest to analyse newer biomarkers to identify persons at risk to develop cardiovascular incidents and cardiovascular death.

Proteomics is a useful technology to identify several proteins at the same time in a single sample, such as plasma. As several authors have reported, proteomics is a useful technology to identify protein isotypes, which cannot be identified using other conventional technologies to determine proteins. Therefore, through proteomics it is possible to understand more in depth the molecular changes that occur during the disease progression. In this regard, proteins and proteins isotypes, identified by proteomics, have not been previously associated with specific CVDs and even drug treatment resistances 10,11.

Taken together, our goal was to analyse, using proteomics, if in apparently healthy individuals there may exist protein biomarkers, particularly those associated with inflammation and thrombosis-coagulation, whose systemic plasma concentrations could be associated with risk of future development of cardiovascular incidents and death.

## Materials and methods

### Study population

As previously reported in detailed, between 1991 and 1996, women aged 45–73 years and men aged 46–73 years, with residency in Malmö (approximately 250,000 habitants), Sweden, were invited by mail and by newspaper advertisement to participate in the Malmö Diet and Cancer Study (MDC) 12,13. Of them, 28,449 persons accepted participation (participation rate 41%), a sample forming the prospective MDC cohort 12,13. From the MDC cohort, 6103 individuals were randomly selected to be studied for the epidemiology of carotid artery disease, and this sample population is referred to as the MDC cardiovascular cohort (MDC-CC) 14. The present study was performed in 41 healthy randomly selected MDC-CC participants who developed CVD (myocardial infarction or ischemic stroke) during a follow-up period of 14.5 ± 3.9 years (see below) and 41 healthy controls (matched for age and gender) who did not. All 82 individuals were free from a history of CVD at inclusion. The investigation developed conforms to the principles outlined in the 1975 Declaration of Helsinki.

All participants provided a medical history and underwent a physical examination and laboratory assessment of cardiovascular risk factors. It assessed the participants for cigarette smoking and diabetes mellitus and measured blood pressure, body mass index, total cholesterol levels, HDL cholesterol levels and serum creatinine levels. Medication use was recorded. For this study, persons who had serum creatinine levels greater than 2.0 mg/dl (176.8 μmol/l) or missing covariates were excluded. Overnight fasting blood samples were obtained in the morning. At inclusion, blood samples were obtained in ethylenediaminetetraacetic acid immediately centrifuged and the plasma aliquoted and stored at −70°C and they were used for the proteomic study.

C-reactive protein was measured by high-sensitivity assay (Roche Diagnostics, Basel, Switzerland). Biochemical parameters were measured at the Department of Clinical Chemistry, Skane University Hospital in Malmö, which was attached to a national standardization and quality control system. The proteomic analysis was performed in blinded form in the Cardiovascular Research Unit in Madrid, Spain which was attached to 9001:2008 International Organization for Standardization certification.

Cardiovascular events were defined as coronary events, or fatal or non-fatal stroke 9. Coronary events were defined as fatal or non-fatal myocardial infarction or death as a result of ischaemic heart disease. Events were identified through linkage of the 10-digit personal identification number of each Swedish citizen with three registries: the Swedish Hospital Discharge Register, the Swedish Cause of Death Register and the Stroke in Malmö register. Myocardial infarction was defined on the basis of *International Classification of Diseases* 9th and 10th Revisions (ICD9 and ICD10) codes 410 and I21 respectively. Death as a result of ischaemic heart disease was defined on the basis of codes 412 and 414 (ICD9) or I22-I23 and I25 (ICD10). Fatal or non-fatal stroke was defined using codes 434 (ICD9) and I63 (ICD10). Follow-up for outcome extended to 1 January 2010. The procedures were in accordance with the institutional guidelines. The Regional Ethical board of Lund University approved the study. All participants had given written informed consent.

### Two-dimensional electrophoresis of plasma proteins, image acquisition and analysis

As previously reported in detail, for two-dimensional electrophoresis (2-DE) 500 μg of total plasma proteins were used 10,15. Plasma samples were loaded on immobilized gradient Immobilized pH Gradient (IPG) strips (18 cm, pH 4-7) and isoelectric focusing was performed with a Protean IEF cell system (Bio-Rad, Hercules, CA, USA). In the second dimension, proteins were resolved on 10% SDS-PAGE gels using a Protean II XL System (Bio-Rad). As previously reported, the gels were then fixed, silver stained during 30 min. and scanned in a UMAX POWERLOOK III Scanner 10,15. One 2-DE gel was performed for each patient and all of the here-identified spots were at least expressed in 70% of the 2-DE gels. The spots were densitometrically analysed by using the Quantity One 4.2.3. software (Bio-Rad). The densitometric intensity of each spot was evaluated after subtracting the background staining of the corresponding gel.

The protein contained in the spots was identified by using mass spectrometry (MS) and tandem mass spectrometry (MS/MS). As previously reported in detail, spots from three different 2-DE gels were excised, digested with trypsin and purified as reported 15. Mass spectrometry and MS/MS analysis were performed in a 4700 Proteomic Analyzer (Applied Biosystem, Old Conneticut path, Frammingham, MA, USA) that operated in a reflector positive mode. Peptides with a signal-to-noise greater than 20 were considered in the Mascot Database for protein identification. To identify the sports, Mascot database 1.9 (http://www.matrixscience.com) was used as an algorithm to match the peptides obtained by MS. As previously reported 15, identifications were accepted based on a tripartite evaluation that takes into account significant molecular weight search (Mowse) scores, spectrum annotation and observed *versus* expected migration on the 2-DE gel. Each spot was identified twice and only plasma proteins and plasma protein isotypes not previously published identified by MS by our group were identified here.

### Dot-blot and Western blot analysis

For dot-blot analysis, 10 μg of total plasma protein from each of the 82 included patients were loaded onto a nitrocellulose membrane as previously reported 16. In brief, nitrocellulose membrane was blocked with 5% (w/v) bovine serum albumin, and incubated with goat polyclonal IgG antibodies against alpha1-antitrypsin (sc-14586; Santa Cruz Bio8technology, Inc. Santa Cruz, CA, USA. dilution 1:1000), vitamin D-binding protein antibody (sc-18705; Santa Cruz Biotechnology, Inc. Santa Cruz, CA, USA. dilution 1:2000) and fibrinogen-γ chain antibody (sc-18032; Santa Cruz Biotechnology, Inc. Santa Cruz, CA, USA. dilution 1:2000). The expression of a constitutive protein, β-actin (A-5441; Sigma-Aldrich, St. Louis, MO, USA, dilution 1:1000), was also determined as a protein load control and to normalize the results. After washing, nitrocellulose membranes were incubated with peroxidase-conjugated goat antimouse IgG for alpha1-antitrypsin, vitamin D-binding protein and fibrinogen-γ chain and peroxidase-conjugated antimouse IgG for β-actin (dilution 1:7500). The blots were developed using enhancing chemiluminiscence reagents (ECL; GE Healthcare, Little Chalfont, Buckinghamshire, UK)

Western blot analysis was further performed. For this purpose, 20 μg of total plasma protein was separated on 15% SDS/PAGE, then blotted onto nitrocellulose membranes, incubated with the same antibodies used for dot-blot analysis and developed using ECL.

### Statistics analysis

SPSS statistical software (version 17.0; SPSS Inc., Chicago, IL, USA) was used for all analyses. Results are expressed as mean ± SEM. Plasma levels were first compared between groups in univariate analyses with Student’s *t*-test or Mann–Whitney’s test, depending on normality, followed by logistic regression analyses adjusted for age and gender (model 1) and additional adjustment for smoking, hypertension, diabetes mellitus, low-density lipoprotein (LDL)-cholesterol, HDL-cholesterol and C-reactive protein (model 2). All statistical tests had a type I error probability of <0.05.

## Results

All participants were followed from the baseline examination until the first cardiovascular event, death or 31 December 2010. Mean follow-up was 14.5 ± 3.9 years.

Table 1 summarizes the clinical characteristics showed by the participants at the baseline examination of MDC-CC (Table 1). Of the 82 participants, 23 (27.7%) died during the 18 years follow-up period and 17 (20.7%) of these deaths were caused by cardiovascular events.

**Table 1:** Baseline clinical characteristics

	Incident CVD (*n* = 41)	No incident CVD (*n* = 41)	*P* value
Age (years)	60 ± 4.4	60 ± 4.8	0.42
Gender *n* (%) females	16 (39)	21 (51)	0.27
Hypertension *n* (%)	35 (85)	30 (73)	0.17
Diabetes *n* (%)	2 (4.9)	0 (0)	0.49
Smoker *n* (%)	14 (34)	8 (20)	0.14
LDL-cholesterol (mM)	4.6 ± 0.8	4.6 ± 1.1	0.89
HDL-cholesterol (mM)	1.2 ± 0.33	1.3 ± 0.31	0.66
Body mass index (kg/m^2^)	26.3 ± 3.5	26.5 ± 4.2	0.83
C-reactive protein (mg/l)	2.8 (1.2–9.9)	1.7 (1.0–2.7)	0.02

Continuous variables are given as means ± SEM except for C-reactive protein which is given as median (interquartile range).

CVD: cardiovascular disease; LDL: low-density lipoprotein; HDL: high-density lipoprotein.

As Table 1 shows, C-reactive plasma levels were within the previously reported normal range. However, circulating C-reactive protein levels were higher in apparently healthy participants but at higher cardiovascular risk as they developed cardiovascular incidents during follow-up (Table 1).

### Expression of plasma proteins and cardiovascular incidents during follow-up

**Figure 1:**
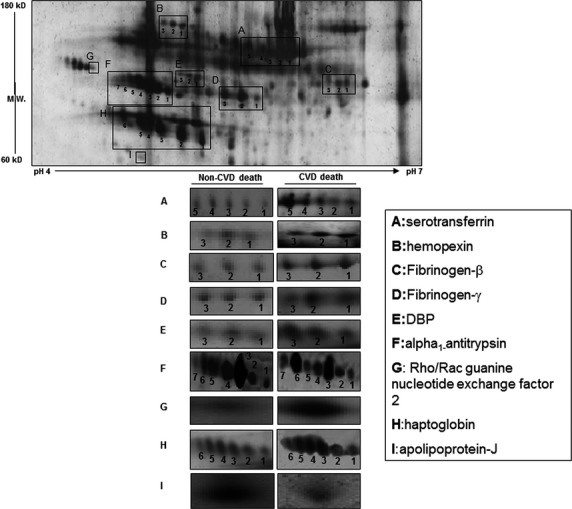
Representative 2-dimensional gel showing the areas contained the different analysed protein isotypes. On the right are shown representative images of each of the protein isotypes determined in participants with cardiovascular disease (CVD) and non-CVD death during follow-up.

**Figure 2:**
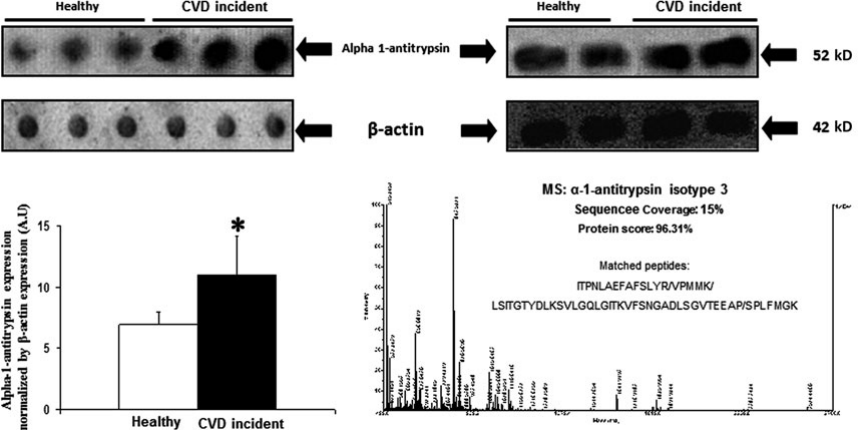
On the left, is shown a representative dot-blot to determine plasma alpha 1-antitrypsin expression. The bar graphs show the densitometric analysis of all dot-blots in arbitrary units (A.U.). The expression of β-actin was used as loading protein control. Densitometric values were normalysed by plasma β-actin expression and are represented as mean ± SEM. *P < 0.05 with respect to participants without cardiovascular incidents during follow-up (healthy). On the upper right side is shown a representative Western blot of alpha-1 antitrypsin showing a single band of apparent molecular weight of 52 kD. On the right down is shown mass spectrometry spectra of alpha 1-antitrypsin isotype 3.

For a better results understanding Figure 1 shows representative 2-DE plasma gel showing the specific areas where spots were analysed. Using 2-DE, seven different α1-antitrypsin isotypes were identified in the plasma but only plasma levels of α1-antitrypsin isotype 3, that was identified by MS (Fig. 2), were higher in participants showing cardiovascular incidents as compared with those without cardiovascular incidents during follow-up (Table 2). Only the spot identified by MS as α1-antitrypsin isotype 3 was significantly higher in the plasma obtained from participants that developed cardiovascular incidents during the follow-up than those without acute cardiovascular episodes (Table 2). Dot-blot analysis showed that total plasma α1-antitrypsin levels were significantly higher in participants developing cardiovascular incidents as compared with those without cardiovascular acute episodes during follow-up (Fig. 2). Western blot analysis supported that the α1-antitrypsin antibody used in dot-blots recognized a single band of apparent molecular weight around 52 kD corresponding to the reported molecular weight for α1-antitrypsin (Fig. 2).

**Table 2:** Expression levels of plasma proteins between incident cases of CVD and control cases

Protein	Incident CVD (A.U), *n* = 41	No incident CVD (A.U), *n* = 41	*P* value
Thrombo-coagulating related proteins			
Fibrinogen β chain			
Isotype 1	96.4 ± 24.1	70.2 ± 15.8	0.575
Isotype 2	223.4 ± 94.9	92.8 ± 21.8	0.742
Isotype 3	164.2 ± 63.5	74.1 ± 13.1	0.354
Fibrinogen γ chain			
Isotype 1	200.7 ± 100.2	109.2 ± 52.8	0.422
Isotype 2	195.6 ± 84.8	72.7 ± 32.9	0.777
Isotype 3	129.7 ± 63.0	57.5 ± 19.0	0.974
Vitamin D-binding protein			
Isotype 1	115.1 ± 36.4	42.16 ± 16.9	0.183
Isotype 2	80.8 ± 24.7	43.3 ± 12.8	0.383
Isotype 3	50.5 ± 14.6	24.2 ± 5.4	0.351
Inflammation-related proteins			
α-1-antitrypsin			
Isotype 1	189.5 ± 53.5	102.4 ± 35.1	0.467
Isotype 2	179.0 ± 69.9	105.6 ± 39.5	0.171
Isotype 3	210.0 ± 65.8	69.2 ± 18.2	0.043
Isotype 4	167.4 ± 52.8	110.4 ± 30.5	0.284
Isotype 5	164.8 ± 60.8	89.3 ± 28.0	0.219
Isotype 6	141.0 ± 39.4	61.0 ± 17.1	0.189
Haptoglobin			
Isotype 1	227.9 ± 54.0	141.6 ± 24.4	0.495
Isotype 2	297.2 ± 54.1	205.2 ± 46.8	0.260
Isotype 3	254.4 ± 47.1	222.3 ± 47.2	0.849
Isotype 4	348.9 ± 80.9	192.0 ± 42.0	0.135
Isotype 5	349.6 ± 110.5	205.7 ± 58.7	0.260
Isotype 6	377.2 ± 169.2	235.1 ± 88.2	0.871
Serotransferrin			
Isotype 1	128.5 ± 28.9	93.6 ± 15.8	0.646
Isotype 2	88.3 ± 19.8	114.9 ± 31.4	0.272
Isotype 3	102.2 ± 21.5	79.8 ± 12.9	0.849
Isotype 4	90.2 ± 20.6	80.7 ± 18.4	0.725
Isotype 5	106.9 ± 31.5	75.0 ± 15.4	0.630
Haemopexin			
Isotype 1	68.8 ± 23.3	45.9 ± 14.0	0.556
Isotype 2	88.4 ± 31.4	44.8 ± 11.5	0.600
Isotype 3	88.9 ± 41.3	36.5 ± 9.1	0.842
Apolipoprotein-J	397.9 ± 59.5	753.5 ± 89.3	0.001
Rho/Rac guanine nucleotide exchange factor 2	320.0 ± 78.0	161.0 ± 30.8	0.062

Results are represented as mean ± SEM.

A.U: Arbitrary units; CVD: cardiovascular disease.

Considered as continuous variable, plasma α1-antitrypsin isotype 3 levels were positively correlated with cardiovascular incidents during the follow-up; however, after adjustment for age and gender the statistical differences disappeared (OR per 1 standard deviation increment: 2.3; 95% CI: 0.94–5.5; *P* = 0.07).

In the 2-DE analysis, plasma apolipoprotein J levels were significantly lower in cases with incident cardiovascular events compared to controls (Table 2). After adjustment for age and gender, this difference remained significant (OR per 1 standard deviation increment: 0.44; 95% CI: 0.25–0.79; *P* = 0.006), however not after additional adjustment for traditional risk factors and C Reactive Protein (CRP) (*P* = 0.077).

The analysis of 2-DE gels also showed that plasma levels of Rho/Rac guanine nucleotide exchange factor also tended to increase in participants showing cardiovascular incidents as compared with those free of them during follow-up. However, it did not reach statistical significance (*P* = 0.062; Table 2). Other proteins identified in plasma associated with inflammation and thrombosis analysed by 2-DE did not reach statistical differences between participants who suffered cardiovascular incidents or not during follow-up (Table 2).

### Expression of plasma proteins and cardiovascular death

Two-dimensional electrophoresis showed that changes in the plasma levels of several plasma proteins were associated with cardiovascular death during follow-up (Table 3). These proteins were mainly associated with inflammation and thrombo-coagulating processes. At the bottom of Figure 1 are shown representative gel images of these plasma proteins in participants with cardiovascular and non-cardiovascular death.

**Table 3:** Expression levels of plasma proteins that reached statistical differences between patients who during the follow-up suffered cardiovascular death or not

Protein	Non-cardiovascular death (A.U), *N* = 65	Cardiovascular death (A.U), *N* = 17	Odds ratio for CVD death (95% confidence interval)*†	Adjusted* *P* value	Adjusted** *P* value
Thrombo-coagulating related proteins					
Fibrinogen β chain					
Isotype 1	63.7 ± 11.0	158 ± 52.5	1.98 (1.12–3.50)	0.019	0.034
Isotype 2	88.2 ± 21.3	425 ± 214	2.40 (1.02–5.66)	0.045	0.062
Isotype 3	61.7 ± 10.2	339 ± 143	4.68 (1.16–18.8)	0.030	0.022
Fibrinogen γ chain					
Isotype 2	79.7 ± 32.2	342 ± 179	1.73 (1.01–2.96)	0.047	0.019
Vitamin D-binding protein					
Isotype 1	41.3 ± 13.0	221 ± 76.9	2.50 (1.27–4.91)	0.008	0.007
Isotype 2	36.7 ± 8.44	159 ± 54.3	3.12 (1.23–7.97)	0.017	0.018
Isotype 3	25.4 ± 6.34	83.0 ± 27.1	2.02 (1.14–3.57)	0.016	0.012
Inflammation-related proteins					
α-1-antitrypsin					
Isotype 3	79.5 ± 23.1	369 ± 131	2.51 (1.22–5.16)	0.012	0.005
Isotype 6	62.1 ± 13.6	250 ± 83.8	2.61 (1.39–4.89)	0.003	0.006
Haptoglobin					
Isotype 3	197 ± 32.7	398 ± 92.1	1.78 (1.09–2.90)	0.022	0.019
Isotype 4	211 ± 45.0	494 ± 131	1.73 (1.05–2.85)	0.032	0.004
Isotype 5	180 ± 39.9	650 ± 247	2.23 (1.17–4.24)	0.015	0.007
Serotransferrin					
Isotype 1	82.0 ± 12.2	222 ± 58.3	2.37 (1.31–4.31)	0.005	0.056
Isotype 3	67.9 ± 8.95	179 ± 44.5	2.67 (1.36–5.26)	0.005	0.078
Isotype 4	67.0 ± 12.1	156 ± 44.5	1.80 (1.10–2.96)	0.019	0.207
Isotype 5	58.9 ± 10.3	214 ± 68.2	2.84 (1.32–6.08)	0.007	0.060
Haemopexin					
Isotype 1	39.0 ± 9.27	128 ± 53.0	1.91 (1.02–3.57)	0.041	0.034
Isotype 2	41.9 ± 9.45	161 ± 69.7	1.99 (1.14–3.48)	0.016	0.009
Apolipoprotein J	690.6 ± 64.9	177.1 ± 51.5	0.024 (0.002–0.27)	0.003	0.004
Rho/Rac guanine nucleotide exchange factor 2	156 ± 25.0	561 ± 163	3.86 (1.63–9.17)	0.002	0.005

* *P* value adjusted for age and gender.

** *P* value adjusted for age, gender, smoking, hypertension, diabetes mellitus, LDL-cholesterol, HDL-cholesterol and C-reactive protein.

† Expressed as per standard deviation increment of protein concentration.

Results are represented as mean ± SEM.

A.U: Arbitrary units; CVD: cardiovascular disease.

The thrombo-coagulant-related proteins associated with cardiovascular death identified in the 2-DE included: fibrinogen β-chain isotypes 1 and 3, fibrinogen-γ-chain isotype 2, vitamin D-binding protein (DBP) isotypes 1,2 and 3.

Among the inflammatory-related proteins associated with cardiovascular death identified in the 2-DE experiments were: apolipoprotein J, α1-antitrypsin isotypes 3 and 6, haptoglobin isotypes 3,4,5 serotransferrin 1,3,4 and 5, haemopexin isotypes 1 and 2, and Rho/Rac guanine nucleotide exchange factor 2 (Table 3). The expression level of these inflammatory and thrombotic-related proteins and protein isotypes was higher in participants that died by cardiovascular event during follow-up than in those with non-cardiovascular death (Table 3).

**Figure 3:**
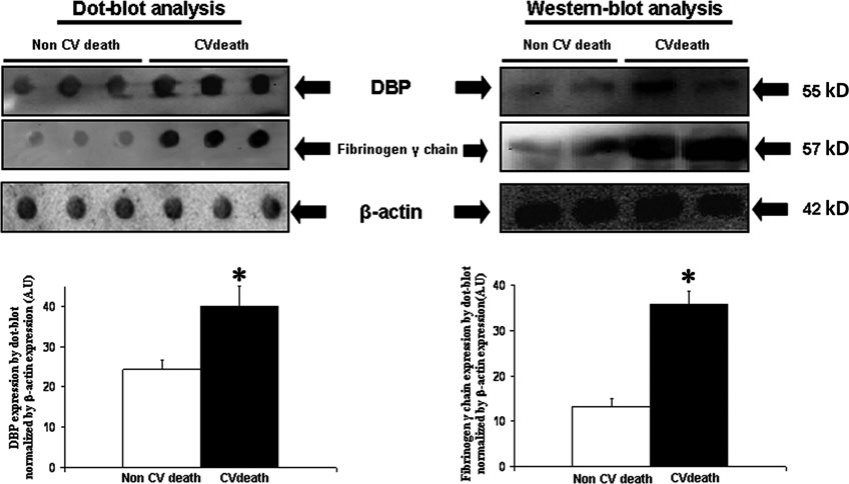
On the upper left side is shown a representative dot-blot to determine plasma vitamin D-binding protein (DBP) and fibrinogen-γ-chain expression in participants with and without cardiovascular death during follow-up. In the images is also shown representatives expression of β-actin in plasma that was used as loading protein control and for to normalize the densitometric analysis in dot-blots. The bar graphs show the densitometric analysis of all dot-blots in arbitrary units (A.U.). Densitometric values were normalysed by plasma β-actin expression and are represented as mean ± SEM. *P < 0.05 with respect to participants with non-cardiovascular death during follow-up. On the upper right side is shown a representative Western-blot showing single bands of 55 and 57 kD of apparent molecular weights corresponding to DBP and fibrinogen-γ-chain respectively.

The association of the increased plasma expression of some of these proteins with future cardiovascular death was also analysed using dot-blot analysis. As Figure 3 shows, dot-blot analysis demonstrated that total expression of both DBP and fibrinogen-γ-chain was significantly higher in participants with cardiovascular death than in those with non-cardiovascular death during follow-up. Western blot analysis supported that the antibodies used in dot-blots recognized single bands of apparent molecular weight around 55 kD for DBP and 57 kD for fibrinogen-γ-chain, corresponding to the reported molecular weight for each of these proteins (Fig. 3).

After age and gender adjustment, plasma levels of several proteins were significantly higher at inclusion in participant who suffered cardiovascular death during follow-up as compared to those with non-cardiovascular death. (Table 3). The plasma expression levels of the other above-mentioned proteins were significantly higher in participants with cardiovascular death as compared with those without cardiovascular death when plasma protein expression levels were adjusted by age, gender, smoking, hypertension, diabetes mellitus, LDL-cholesterol, HDL-cholesterol and C-reactive protein (Table 3). The only proteins for which the statistical differences between participants with and without cardiovascular death disappeared when adjustment for conventional risk factors and C-reactive protein were serotransferrin isotypes 1,3,4 and 5 (Table 3).

As mentioned, most of the plasma proteins associated with cardiovascular death during follow-up showed higher levels in these participants than in the participants with non-cardiovascular death. However, it was also identified by 2-DE a plasma protein whose expression was lower in participants suffering cardiovascular death during follow-up. This protein was apolipoprotein J (Fig. 1 and Table 3). Plasma apolipoprotein J expression remained lower in participants that during follow-up died by cardiovascular event as compared with those without cardiovascular death when values were adjusted by age and gender and when they were adjusted by age, gender, smoking, hypertension, diabetes mellitus, LDL-cholesterol, HDL-cholesterol and C-reactive protein (Table 3).

## Discussion

One of the most important challenges in the prevention of an acute cardiovascular ischaemic event and the risk of cardiovascular death is the identification of biomarkers that may improve the early detection of the ischaemic process before an irreversible injury occurs. The present investigation was based on the analysis of a panel of plasma proteins to try to identify whether some of them could be associated with the prediction of risk of cardiovascular incidents and cardiovascular death, above and beyond conventional risk factors. The study was performed in a Swedish population-based cohort with longitudinal follow-up.

For incidence of CVD, the main finding was that increased plasma levels of α1-antitrypsin might be a predictor of cardiovascular events. The 2-DE suggested that probably this increase in α1-antitrypsin plasma levels were related to α1-antitrypsin isotype 3.

α1-antitrypsin belongs to a family of acute−phase inflammatory proteins and its most known function is the inhibition of proteolytic enzymes. Moreover, α1-antitrypsin was also associated with thrombosis and coagulating activity in addition to acute coronary events 10,17,18. However, after adjusting by age and gender, the significant difference in the plasma expression of α1-antitrypsin isotype 3 between individuals that during follow-up will suffer a cardiovascular incident was lost, which diminished the interest of this finding which should be taken with caution.

An interesting finding was that apolipoprotein J showed lower plasma expression levels in participants with cardiovascular incidents than those without that during follow-up suggesting apolipoprotein J as predictor of cardiovascular events. It was not dependent of age and gender. Apolipoprotein J, also known as clusterin, is expressed in many tissues, and is a component of HDLs. There are experimental evidence that apolipoprotein J may has anti-atherogenic properties through its effects on cholesterol transport, smooth muscle cell proliferation and lipid peroxidation 19. Indeed, apolipoprotein J infusion reduced both infarct size and death in rat undergoing experimental myocardial infarction 20. In this regard, the fact that statistical significance was lost after adjustment by conventional risk factor and C-reactive protein suggest the impact of such factors in the reduced circulating expression levels of apolipoprotein J observed in the participants suffering cardiovascular incidents during follow-up.

The most striking observation from our findings was probably that the plasma level of many proteins and protein isotypes associated with thrombosis and inflammation were independently associated with risk of cardiovascular death. In this regard, participant who suffered cardiovascular death during follow-up showed an increased expression of plasma proteins w hen they were apparently healthy. Accordingly, previous works have showed that increased levels of other more classical inflammatory and thrombo-coagulating-sensitive plasma proteins are linked to increased risk of vascular complications and were even identified as predictors of near-term mortality related to cardiovascular incidents 6,21.

In our study, specific isotypes of fibrinogen β- and -γ chains were found increased in healthy individuals that suffered cardiovascular death during follow-up. Fibrinogen is a 340 kD complex hexamer containing two sets of three chains (α, β and γ) 22. Both β- and -γ chains were involved to bind fibrin to platelets 23. Interestingly, it was reported that increase in plasma fibrinogen in patients with stable intermittent claudication predicted a nearly twofold increase in the probability of death within the next 6 years 24. In addition, fibrinogen was also identified as an independent predictor of mortality in patients with coronary heart disease, and for a broad spectrum of diseases in elderly men. However, the predictive value of fibrinogen on mortality was mainly attributed to its inflammatory property better than to its thrombotic role 25,26.

Plasma levels of three DBP isotypes were also associated with future cardiovascular death. Vitamin D-binding protein has been associated with both inflammatory and thrombotic mechanisms 27,28. Interestingly, increased plasma levels of DBP were also associated with platelet resistance to Aspirin 29.

Taken together, the importance of the present results is that plasma levels of specific isotypes of two thrombotic-related proteins, fibrinogen and DBP, were associated with cardiovascular death in individuals who have already not manifested CVD when the plasma sample was obtained.

Another group of proteins whose plasma expression was associated with future cardiovascular death were inflammatory-related proteins. At present, modest results had been obtained after the analysis of more traditional inflammatory biomarkers, *i.e*. cytokines to predict cardiovascular death. In addition, most of the previous studies had been followed in patients with previous CVD 30,31. In the present study higher plasma levels of two α1-antitrypsin isotypes, three haptoglobin isotypes and two haemopexin isotypes were associated with future cardiovascular death. Moreover, lower plasma levels of apolipoprotein J were also associated with cardiovascular death during follow-up.

There are several ambiguities concerning the action of α1-antitrypsin in the development of vascular complications. In this regard, α1-antitrypsin concentration was reported increased in human aortic atherosclerotic lesions 32, however, other works have demonstrated reduced blood pressure and stiffness in the abdominal aorta of individuals with α1-antitrypsin deficiency 33,34. These apparently reported contradictory results could be influenced by a heterogeneous specific function for each α1-antitrypsin isotype. In this study, only α1-antitrypsin isotypes 3 and 6 were found increased in the patients that suffered cardiovascular death.

The increased plasma levels of three specific haptoglobin and two haemopexin isotypes were also associated with future cardiovascular death. Haptoglobin and haemopexin are two close-related acute phase inflammatory proteins. Working together, haptoglobin and haemopexin may act as detoxificators of haeme-binding proteins and participate in the Fenton reaction to produce reactive oxygen species causing cell injury. Therefore, both haptoglobin and haemopexin have anti-oxidative properties 35. This observation may seem paradoxical as isotypes from two hypothetical cell-protecting proteins were increased in the plasma of patients that will die for a cardiovascular cause. However, it may be explained by the possible existence of a very premature increase in oxidative stress. Therefore, the organism will try to counterbalance this silent oxidative stress damage enhancing the expression of antioxidant-related proteins, such as haemopexin and haptoglobin. In this regard, a strong association between the haptoglobin phenotypes and the risk of developing premature coronary and peripheral vascular diseases has been previously reported 36,37.

Plasma levels of Rho/Rac guanine nucleotide exchange factor 2 were also associated with future cardiovascular death. Both *in vitro* and *in vivo* studies have suggested the involvement of numerous downstream targets of Rho and Rac signalling, including transcription factors *i.e*. NF-κB, and other inflammatory-related transcription factors, further to be a regulator of: cytoskeletal organization, including myofilament proteins, ion channels and reactive oxygen species generation 38. However, the *in vivo* effects of Rho/Rac activation remain to be established.

The apolipoprotein J was the only protein whose expression was found reduced in participants who suffered cardiovascular death during follow-up as compared to participants with non-cardiovascular death. As above-mentioned, in experimental animals apolipoprotein J was associated with the reduction in death after myocardial infarction 20. Moreover, it was also reported that apolipoprotein J may have anti-inflammatory properties 39 and therefore, its reduction may be contributing to the here observed apparent higher pro-inflammatory state in participants that will suffer cardiovascular death during follow-up.

Association between plasma levels of all these plasma proteins and cardiovascular death was independent of age, gender, conventional risk factors and plasma C-reactive protein levels.

The fact that several proteins showed an increased expression in the plasma from participants with cardiovascular death, but at least apolipoprotein J has lower expression in these participants support the specificity of the observed findings.

In summary, in our knowledge this is one of the first proteomic studies showing that specific protein isotypes related to thrombo-coagulation and inflammation may predict, at long-term in apparently healthy people, risk of cardiovascular death. Interestingly, the association of most of these protein isotypes with cardiovascular death were independent of conventional risk factors, age, gender and even C-reactive protein levels. The fact that increased plasma levels of several different and specific protein isotypes were independently associated with the risk of cardiovascular death might reflect the complexity of all the facets of cardiovascular death.

Several limitations of our analysis deserve comment. It used the national in-patient and cause-of-death registers to ascertain cardiovascular events. This method to ascertain cardiovascular end-points have been validated and found to have good accuracy for cardiovascular end-points 40. The present work does not include ‘non-major’ cardiovascular events (angina, intermittent claudication or transient ischaemic attack) as cardiovascular incidents. The reason for not including them was that although the register based end-point retrieval could be highly validated for myocardial infarction and stroke, this has not been the case for angina, intermittent claudication and transitory ischemic attack. Thus, our participants cannot be viewed strictly for studying primary prevention.

In the study, circulating C-reactive protein levels were used as a covariate in the logistic regression model. Future studies should compare the here identified plasma proteins that were associated with cardiovascular death with other more conventional published protein biomarkers for cardiovascular death even C-reactive protein by itself. However, C-reactive proteins and others considered as conventional biomarkers have more often been associated with cardiovascular risk better than to cardiovascular death 41,42.

It should be stressed that several tests were performed and the presented *P* values are not corrected for multiple comparisons. Given the inter-correlation between several of the markers, and thus dependency between some of the tests, a Bonferroni correction would seem too conservative. In any case, it is important to replicate our findings in an independent population before drawing firm conclusions.

## Conclusions

The here reported data suggest that increased plasma levels of several plasma proteins and protein isotypes related to inflammation and thrombo-coagulating phenomena were associated with the risk of future cardiovascular death. Proteomic analysis may provide a specific and suitable alternative to obtain global information about changes in the plasma levels of several biomarkers and makes possible the detection of different protein isotypes that are not identified by conventional measure of the circulating biomarkers. Moreover, it could be useful to understand and know more in depth the molecular mechanisms involved in cardiovascular death.
